# COVID-19 vaccination among health care workers in Finland: coverage, perceptions and attitudes

**DOI:** 10.1177/14034948231203779

**Published:** 2024-01-03

**Authors:** Aleksi Hämäläinen, Riitta-Liisa Patovirta, Sakari Vuorinen, Jaana Leppäaho-Lakka, Sanna Kilpinen, Jennifer Sieberns, Eija Ruotsalainen, Irma Koivula, Sari Hämäläinen

**Affiliations:** 1 University of Eastern Finland Institute of Clinical Medicine, Kuopio, North Savo, Finland; 2 Kuopio University Hospital, Kuopio, North Savo, Finland; 3 Mikkeli Central Hospital, Mikkeli, South Savo, Finland; 4 Hospital Nova of Central Finland, Jyväskylä, Central Finland, Finland; 5 North Karelia Central Hospital, Joensuu, North Karelia, Finland; 6 Savonlinna Central Hospital, Savonlinna, South Savo, Finland

**Keywords:** COVID-19, COVID-19 vaccination, health care workers, mandatory vaccination, vaccination, vaccine hesitancy

## Abstract

**Aims:**

In this study, we examined the voluntary COVID-19 vaccine coverage among health care workers (HCWs) working in close patient contact. HCWs’ beliefs about COVID-19 infection, their opinions of vaccination and reasons for having or declining the COVID-19 vaccination were also evaluated.

**Methods:**

In October 2021, a cross-sectional observational study was carried out in five hospitals in Central and Eastern Finland. The anonymous and voluntary survey was targeted at 5120 doctors and nurses working in close patient contact.

**Results:**

Some 1837 responses were included in the study. Ninety-seven per cent of the respondents had received at least one COVID-19 vaccine and 68% of the respondents agreed that all HCWs working in close patient contact should be vaccinated against COVID-19. Vaccination coverage and support for vaccination were higher among older HCWs and doctors. HCWs’ main reasons for having the COVID vaccine were willingness to protect themselves, their family and their patients from COVID-19. Concerns about adverse reactions to the COVID-19 vaccine was the main reason for declining it.

**Conclusions:**

**The overall COVID-19 vaccination coverage and support for vaccinations among HCWs working in close patient contact were high without actual mandatory policies being introduced. Prioritising HCWs for COVID-19 vaccinations and widespread vaccine availability, as well as low general vaccine hesitancy and high seasonal influenza vaccination coverage among the study population were check marks in achieving high COVID-19 vaccination coverage rapidly.**

## Background

COVID-19 rapidly became a major public health crisis, affecting 677 million individuals and causing 6.9 million deaths globally by March 2023 [1]. Vaccines are efficient in preventing SARS-CoV-2 infection and reduce the amount of viral load, risk of febrile symptoms and duration of the illness [2]. COVID-19 vaccine uptake was therefore an important challenge to address. In a recent systematic review, the highest COVID-19 vaccine acceptance rates among the general population were found in Ecuador (97.0%), Malaysia (94.3%) and Indonesia (93.3%). In Europe, the vaccine acceptance rate ranged from 27.0% to 91.5% [3].

Health care workers (HCWs) play a crucial role in the decision making about immunisation. HCWs’ recommendation for vaccination was one of the main factors influencing vaccine uptake in elderly patients [4]. In low- and middle-income countries, HCWs were the most trusted source of guidance about COVID-19 vaccines [5], and more likely to accept a COVID-19 vaccine than the general population [6,7]. In another systematic review, the overall COVID-19 vaccination coverage among HCWs was 77.3% (ranging from 17.9% to 96.0%), with higher vaccine coverage found in studies conducted in North America (85.6%) than in studies conducted in Asia (79.5%), Europe (72.8%) and Africa (65.6%) [8]. In studies conducted in Europe, the vaccination coverage ranged from 17.9% in Georgia to 92% in Norway [9,10].

Among HCWs, factors associated with higher COVID-19 vaccine uptake include male gender, older age, working as a physician, previous year’s history of influenza vaccine uptake, and perceived individual risk of COVID-19 [11]. The main concerns regarding the COVID-19 vaccine have been issues related to the vaccine’s safety, fear of side effects and doubts about vaccine efficacy and effectiveness [11].

In Finland, COVID-19 affected up to 1.5 million individuals and had caused 9000 deaths by March 2023 [1]. COVID-19 vaccinations became available in Finland at the end of December 2020. The first vaccines used were Pfizer’s Comirnaty®, Moderna’s Spikevax® messenger RNA (mRNA) and AstraZeneca’s adenovirus vector vaccine, Vaxzevria®, with significantly greater quantities of the Comirnaty® vaccine being administered at the beginning of 2021 due to its availability. In March 2021, the Finnish Institute for Health and Welfare temporarily suspended the use of Vaxzevria® after The Finnish Medicines Agency, Fimea, reported two cases of cerebral venous sinus thrombosis within 4–10 days of receiving the vaccine [12]. In April 2021, after the notice from the European Medicines Agency that unusual blood clots with low blood platelets should be listed as a very rare side effect of Vaxzevria® [13], COVID-19 vaccinations continued only with mRNA vaccines among those under the age of 65. In Finland, HCWs were prioritised for COVID-19 vaccinations. Those working with patients with confirmed or suspected COVID-19 disease, HCWs working in urgent care and those working in nursing homes were the first to receive the COVID-19 vaccines. From June 2021, other HCWs were vaccinated among the general population, once the elderly over 70 years of age and patients belonging to risk groups susceptible to severe consequences from COVID-19 had been vaccinated. By October 2021, the first-dose CO-VID-19 vaccination coverage among all Finnish residents over 18 years of age was 81.9% [14]. The vaccines were free of charge and voluntary for all HCWs and were mainly administered at employees’ workplaces during working hours.

## Aims

In this study, we examined the COVID-19 vaccine coverage among HCWs working in close patient contact in Eastern and Central Finland. We also examined HCWs’ opinions and beliefs about COVID-19 infection and vaccination and explored their reasons for having or declining the COVID-19 vaccination.

## Methods

### Data collection

This cross-sectional observational study was carried out in five hospitals in Central and Eastern Finland. The participating hospitals were Kuopio University Hospital in Kuopio, Central Finland Central Hospital in Jyväskylä, North Karelia Central Hospital in Joensuu, Mikkeli Central Hospital in Mikkeli and Savonlinna Central Hospital in Savonlinna. The five hospitals provide secondary and tertiary care services to approximately 805,000 citizens in Central and Eastern Finland. At the start of the survey, a total of 5120 nurses and doctors worked in close patient contact in the five study hospitals according to each hospital’s human resources department. The study was conducted in October 2021 within a year of the availability of the COVID-19 vaccines, when all HCWs in Finland had been able to have the first COVID-19 vaccine voluntarily. The study conducted was the first in Finland and the second among all the Nordic countries. The anonymous and voluntary survey was created using the Surveypal tool, and the link to the online survey was sent to recipients via email together with a cover letter. The emails were sent to HCWs using the email distribution lists of nurses and doctors obtained from the hospital managements of each participating hospital. A notification email and link to the survey were sent to HCWs 1 week after the first email. The survey was closed at the end of October 2021.

### Survey items

The survey gathered information on gender, age, profession (doctor, nurse), workplace, current first-dose COVID-19 vaccination status, opinions about the severity of the COVID-19 infection both to themselves and persons belonging to risk groups for severe COVID-19 infection (e.g. aged 70 years or older, obese, heart or lung diseases, liver or kidney deficiency, diabetes, and immunodeficiency), the effect of the type of the vaccine (vaccine technology and the manufacturer of the vaccine) on vaccine uptake, their opinions on whether all HCWs working in close patient contact should be vaccinated against COVID-19 and their personal reasons for either COVID-19 vaccine uptake or declination.

### Data analysis

The data was converted from the Surveypal program to an SPSS file, and data analysis was completed using SPSS (IBM SPSS Statistics for Windows, Version 28.0. Armonk, NY: IBM Corp). The general characteristics were summarised using descriptive statistics. Statistical analyses used the chi-square (χ^2^) test for comparison between categorical variables. Fisher’s exact test was used instead of the chi-square test to analyse categorical variables, if any cells had a low (<5) minimum expected count. In questions with more than two possible answers, the variable analysed was compared with other groups combined. Results with a *p*-value lower than 0.05 were considered to be statistically significant.

### Ethical approval

This study was conducted according to the principles expressed in the Declaration of Helsinki [15] and approved by local ethics committees in all five study hospitals (Kuopio University Hospital 1393/2021 1.7.2021, Central Finland Central Hospital 48646/2021 29.7.2021, North Karelia Central Hospital 31/2021 30.7.2021, Mikkeli Central Hospital 36/2021 2.7.2021, Savonlinna Central Hospital 48800/2021 30.6.2021).

## Results

Some 2028 responses to the questionnaire were received. Ninety responses from HCWs who were not working in close patient contact and from em-ployees other than nurses and doctors were excluded. A further 101 surveys that were not filled in completely were also excluded, leaving 1837 study responses – a response rate of 35.9%. Most of the respondents were female (1467, 79.9%). The age distribution of the respondents consisted of under 30 years: 232 (12.6%), between 30 and 50 years: 968 (52.7%) and over 50 years old: 637 (34.7%). Doctors constituted 477 of the respondents (26.0%) and 1360 (74.0%) were nurses.

The results for COVID-19 vaccination status and opinions about COVID-19 among all respondents are presented in Table I. Most of the respondents (97.1%) had received at least one COVID-19 vaccine. Altogether, 1053 (57.3%) of the respondents considered COVID-19 to be a serious illness to themselves, and 1815 (98.8%) considered it to be a serious illness to persons belonging to risk groups. The type of vaccine being offered had no effect on vaccination rates among 1295 (70.5%) of the respondents. Over half of the respondents (67.6%) agreed that the COVID-19 vaccine should be administered to all HCWs working in close patient contact. Vaccination coverage was slightly higher among doctors than nurses (99.2% vs. 96.4%, *p* = 0.002). The type of vaccine being administered had a greater influence on nurses’ than on doctors’ decision to have the vaccine (26.7% vs. 13.8%, *p* < 0.001). Doctors were more supportive than nurses of the statement that all HCWs working in close patient contact should be vaccinated against COVID-19 (86.8% vs. 60.8%, *p* < 0.001).

**Table I.** COVID-19 vaccination status and opinions about COVID-19 among all respondents and by profession. *P*-values were calculated using the chi-square (χ^2^) test and Fisher’s exact test.

	All respondents	Doctors	Nurses	*P*-value
	*n* = 1837	*n* = 477	*n* = 1360	
	*n* (% in the category)	*n* (% in the category)	*n* (% in the category)	
Have you received at least one COVID-19 vaccine?				
Yes	1784 (97.1)	473 (99.2)	1311 (96.4)	0.002
No	53 (2.9)	4 (0.8)	49 (3.6)	
Do you consider COVID-19 to be a serious disease to you personally?				
Yes	1053 (57.3)	257 (53.9)	796 (58.5)	0.077
No	440 (24.0)	147 (30.8)	293 (21.5)	<0.001
Uncertain	344 (18.7)	73 (15.3)	271 (19.9)	0.026
Do you consider COVID-19 to be a serious disease to persons belonging to risk groups?				
Yes	1815 (98.8)	473 (99.2)	1342 (98.7)	0.402
No	9 (0.5)	2 (0.4)	7 (0.5)	0.574*
Uncertain	13 (0.7)	2 (0.4)	11 (0.8)	0.303*
Has the type of the vaccine influenced to your decision to have the vaccine?				
Yes	429 (23.4)	66 (13.8)	363 (26.7)	<0.001
No	1295 (70.5)	393 (82.4)	902 (66.3)	<0.001
Uncertain	113 (6.2)	18 (3.8)	95 (7.0)	0.006
Do you agree that all HCWs working in close patient contact should be vaccinated against COVID-19?				
Yes	1241 (67.6)	414 (86.8)	827 (60.8)	<0.001
No	370 (20.1)	30 (6.3)	340 (25.0)	<0.001
Uncertain	226 (12.3)	33 (6.9)	193 (14.2)	<0.001

*Note*:*Calculated using Fischer’s exact test.

The impact of gender and age on vaccine uptake and opinions about COVID-19 are presented in Supplementary Table 1. Females considered COVID-19 to be a serious illness for themselves more often than males did (58.6% vs. 52.4%, *p* = 0.033), and the type of vaccine had a greater influence on the female respondents’ decision to have the vaccine compared with the male respondents (24.7% vs. 17.8%, *p* = 0.005). Vaccine uptake was higher among HCWs >50 years old (99.1%, *p* < 0.001) than those who were under 30 (95.7%) or 30–50 years old (96.2%). The HCWs’ perception that COVID-19 was a serious disease to them personally also increased with age (*p* < 0.001). The same was true for the support for vaccinations being given to all HCWs working in close patient contact: 52.6% of those <30 years, 64.2% of 30–50 year olds and 78.2% of those >50 years (*p* = 0.001).

A comparison between vaccinated and not vaccinated HCWs’ opinions are presented in Table II. Those who were vaccinated more frequently considered COVID-19 to be a serious illness to them personally (58.4% vs. 22.6%, *p* < 0.001) and to patients belonging to risk groups (99.2% vs. 84.9%, *p* < 0.001). The type of vaccine had no effect on vaccination uptake.

**Table II.** Opinions about COVID-19 by vaccination status. *P*-values were calculated with the chi-square (χ^2^) test and Fisher’s exact test.

	Vaccinated	Not vaccinated	*P*-value
	*n* = 1784	*n* = 53	
	*n* (% in the category)	*n* (% in the category)	
Do you consider COVID-19 as a seriousdisease to you personally?			
Yes	1041 (58.4)	12 (22.6)	<0.001
No	414 (23.2)	26 (49.1)	<0.001
Uncertain	329 (18.4)	15 (28.3)	0.070
Do you consider COVID-19 to be a serious disease to persons belonging to risk groups?			
Yes	1770 (99.2)	45 (84.9)	<0.001*
No	4 (0.2)	5 (9.4)	<0.001*
Uncertain	10 (0.6)	3 (5.7)	0.005*
Has the type of the vaccine influenced to your decision to have the vaccine?			
Yes	414 (23.2)	15 (28.3)	0.388
No	1263 (70.8)	32 (60.4)	0.101
Uncertain	107 (6.0)	6 (11.3)	0.103*
Do you agree that all HCWs working in close patient contact should be vaccinated against COVID-19?			
Yes	1241 (69.6)	0 (0.0)	<0.001
No	320 (17.9)	50 (94.3)	<0.001
Uncertain	223 (12.5)	3 (5.7)	0.135

*Note*:*Calculated with Fischer’s exact test.

A comparison of the reasons for having or declining the COVID-19 vaccine between vaccinated and unvaccinated respondents is presented in Table III. Note that the vaccinated HCWs answered the questions pertaining to why they would not take the COVID-19 vaccine and vice versa. Those vaccinated appreciated the self-protective effects of the vaccination more than the unvaccinated HCWs did for themselves (95.4% vs. 35.8%, *p* < 0.001), their patients (89.2% vs. 28.3%, *p* < 0.001), their family members (94.1% vs. 32.1%, *p* < 0.001) and their colleagues (85.2% vs. 30.2%, *p* < 0.001). Unvaccinated HCWs were significantly more concerned about the vaccine’s potential side effects (84.9% vs. 12.6%, *p* < 0.001), safety (62.3% vs. 2.0%, *p* < 0.001) and efficacy (50.9% vs. 1.2%, *p* < 0.001) than vaccinated HCWs. Disliking external pressure to have the vaccine was also significantly more common among unvaccinated than vaccinated HCWs (58.5% vs. 5.4%, *p* < 0.001). Some unvaccinated HCWs believed that they did not need the vaccination because of their good general health (28.3% vs. 0.8%, *p* < 0.001) and did not consider COVID-19 to be a serious enough illness to have the vaccine (28.3% vs. 0.2%, *p* < 0.001) compared with vaccinated HCWs. Avoidance of all vaccines and medications was significantly more common among the unvaccinated than vaccinated HCWs (13.2% vs. 0.2%, *p* < 0.001). The belief that the COVID-19 vaccination could cause COVID-19 infection was also significantly more common among unvaccinated than vaccinated HCWs (3.8% vs. 0.2%, *p* = 0.011).

**Table III.** Reasons for having or declining the COVID-19 vaccination between vaccinated and unvaccinated HCWs.

Not vaccinated	All respondents	Vaccinated	Not vaccinated	*P*-value
*n* = 1784	*n* = 1837	*n* = 1784	*n* = 53	
*n* (% in the category)	*n* (% in the category)	*n* (% in the category)	*n* (% in the category)	
I would take the COVID-19 vaccine because . . .				
I want to protect myself from COVID-19	1721 (93.7)	1702 (95.4)	19 (35.8)	<0.001*
I want to protect my patients from COVID-19	1606 (87.4)	1591 (89.2)	15 (28.3)	<0.001
I want to protect my family from COVID-19	1696 (92.3)	1679 (94.1)	17 (32.1)	<0.001*
I want to protect my colleagues from COVID-19	1536 (83.6)	1520 (85.2)	16 (30.2)	<0.001
I want to avoid work absences	740 (40.3)	733 (41.1)	7 (13.2)	<0.001
My supervisor recommended I had the vaccine	90 (4.9)	90 (5.0)	0 (0.0)	0.067*
My colleague recommended I had the vaccine	42 (2.3)	42 (2.4)	0 (0.0)	0.288*
Getting the vaccine is easy	978 (53.2)	975 (54.7)	3 (5.7)	<0.001
The vaccine is free	929 (50.6)	922 (51.7)	7 (13.2)	<0.001
I would not take the COVID-19 vaccine, because . . .				
I am concerned about the vaccine’s adverse reactions	269 (14.6)	224 (12.6)	45 (84.9)	<0.001
I have a medical contraindication, so I can’t have the vaccine	37 (2.0)	34 (1.9)	3 (5.7)	0.089*
I am healthy, so I don’t need the vaccine	30 (1.6)	15 (0.8)	15 (28.3)	<0.001*
I don’t believe I’ll get COVID-19, so I don’t need the vaccine	4 (0.2)	3 (0.2)	1 (1.9)	0.111*
I don’t consider COVID-19 to be a serious enough illness to have the vaccine	18 (1.0)	3 (0.2)	15 (28.3)	<0.001*
I doubt the vaccine’s efficacy in preventing COVID-19	48 (2.6)	21 (1.2)	27 (50.9)	<0.001*
I don’t consider the vaccine to be safe	69 (3.8)	36 (2.0)	33 (62.3)	<0.001*
Getting the vaccine is difficult or takes too much time	5 (0.3)	4 (0.2)	1 (1.9)	0.136*
I dislike having injections	15 (0.8)	15 (0.8)	0 (0.0)	0.643*
I don’t like being pressured to take the vaccine	127 (6.9)	96 (5.4)	31 (58.5)	<0.001*
I could get COVID-19 as a result of taking the vaccine	6 (0.3)	4 (0.2)	2 (3.8)	0.011*
I try to avoid all vaccines and medications	11 (0.6)	4 (0.2)	7 (13.2)	<0.001*

*Note*: All respondents were able to answer all the questions, regardless of whether they had received or declined a COVID-19 vaccination. *P*-values were calculated using the chi-square (χ^2^) test and Fisher’s exact test.

* Calculated using Fischer’s exact test.

## Discussion

We examined both COVID-19 vaccine coverage and opinions about vaccination among HCWs working in close patient contact in five hospitals in Central and Eastern Finland. The voluntary vaccination coverage reaching 97.1% in our survey was higher than in earlier studies [8]. Self-reported coverage was in the same range as the seasonal influenza vaccination coverage among Finnish HCWs, despite vaccination against influenza being legally determined [16]. High vaccine coverage was achieved rapidly, within a year after COVID-19 vaccines became available.

Several reasons might explain the HCWs’ high voluntary COVID-19 vaccination uptake found in our study. Widespread vaccine availability and free vaccination were important factors both in relation to the vaccination uptake in our study with COVID-19 and in earlier studies focusing on seasonal influenza [17]. In Finland, vaccinations were organised by municipalities and offered free of charge, and the rapid launch of the vaccinations was supported by the existing vaccination capacity in municipalities [18]. Although during the time frame of our study the difference in vaccine coverage between HCWs and the general population was similar to that found in earlier studies [6,7], prioritising HCWs in COVID-19 vaccinations probably accelerated the HCWs’ vaccinations in early 2021 when fewer COVID-19 vaccines were available. Due to prioritisation and the rapid launch of the vaccinations, many of the Finnish HCWs received the first COVID-19 vaccine before the Finnish Institute for Health and Welfare suspended the use of AstraZeneca’s COVID-19 vaccine, Vaxzevria®. This caused extensive discussion in the Finnish media about the safety of COVID-19 vaccines. In earlier studies, HCWs vaccinated against seasonal influenza in previous years were found to be more likely to accept the COVID-19 vaccine compared with those not vaccinated [11]. Seasonal influenza vaccination coverage was high among HCWs in our study due to it being legally determined [16], which could be one of the reasons behind the high COVID-19 vaccination coverage achieved. Further-more, general vaccine hesitancy seemed to be low among our study population, since only a few respondents said they try to avoid all vaccinations and medications. Compared with previous studies, there was no difference in the perceived individual risk of COVID-19 infection among HCWs, which would explain the higher vaccine coverage achieved in our study [19,20].

Self-reported vaccination coverage was higher among doctors than nurses. Gender had no effect, but higher age was associated with higher vaccination coverage, as in earlier studies [11]. Respondents over 50 years of age perceived greater individual risk from COVID-19 than the younger respondents in our study, which might explain the higher vaccination coverage among older HCWs. The majority of HCWs in our study agreed that all HCWs working in patient contact should be vaccinated against COVID-19. This support was higher than the support for statutory seasonal influenza vaccination found in our similar study [16]. The HCWs’ individual reasons for having or declining the vaccine were mainly those found in earlier studies [11]. The findings were also similar to research on seasonal influenza vaccines [17], even though COVID-19 vaccines were developed rapidly and, during the study period, information about the safety and efficacy of the COVID-19 vaccines compared with the seasonal influenza vaccine was limited. The protection provided by the COVID-19 vaccine was clearly the most important reason for having the vaccine, but convenient access and being free of charge were also valued, indicating that employers’ inputs also had an impact on vaccine uptake. External recommendations appeared to have little effect, reinforcing the idea that the decision to have the vaccine or not was based on the employee’s own values.

Among those not vaccinated, only the protective effects of the vaccination showed some importance in the reasons for taking the vaccine. For the majority of the HCWs, both vaccinated and unvaccinated, the manufacturer or technology of the vaccine had no effect on vaccination uptake. Nonetheless, concerns about potential adverse reactions to the vaccine and about safety were the main reason for refusing to take the COVID-19 vaccine among HCWs not vaccinated. The results indicated that studies confirming the effectiveness and safety of the COVID-19 vaccines could change the opinions of non-vaccinated HCWs to take the vaccine. Many of the HCWs declining the vaccine did not consider COVID-19 to be a serious enough infection to warrant it, or considered themselves to be sufficiently healthy to not need the vaccine. However, the self-perceived low risk itself seemed to be a lesser factor in refusing the vaccine than HCWs’ fears about the vaccine’s safety and potential side effects.

At the end of 2021, the Finnish Parliament accepted a temporary amendment to the Act of Communicable Diseases [21]. Preparation of the amendment began after the European Centre for Disease Prevention and Control had published the 17th update of the ‘Assessment of the current SARS-CoV-2 epidemiological situation in the EU/EEA’ on 24 November 2021 [22]. The amendment was submitted to the Finnish Parliament on 8 December 2021 [21] and implemented on 1 January 2022, remaining in force until 31 December 2022. According to the temporary amendment, only HCWs who had been fully vaccinated against COVID-19 or HCWs who had had laboratory-confirmed COVID-19 infection within the past 6 months were allowed close-contract treatment of patients susceptible to severe consequences from COVID-19. HCWs with a medical contraindication to the COVID-19 vaccine were able to work if they had had a negative COVID-19 test result carried out not more than 72 h before entering the workplace. HCWs without such protection against COVID-19 would be transferred to another unit or to a position that did not involve patient contact.

This study has several strengths and limitations. With 1837 responses, this is one of the largest survey studies examining HCWs’ COVID-19 vaccination coverage and attitudes towards COVID-19 vaccination [8]. Within the 5120 doctors and nurses contacted about participating in our study, the response rate was low (35.9%), yet sufficient for a quantitative study. However, due to the response rate, selection bias cannot be ruled out, as HCWs who did not have the vaccine may have been less likely to complete the survey. However, our study response rate did not differ from other similar survey studies [8]. The study was conducted in five hospitals providing secondary and tertiary care services in Central and Eastern Finland, therefore, it does not represent primary care or Finland nationwide. The survey was completed anonymously to facilitate collation of reliable data, we were therefore not able to confirm the respondents’ vaccination status from medical records. We researched doctors and nurses working in close patient contact, hence this study and its findings cannot be applied to other professions or even to HCWs not working in close patient contact.

## Conclusions

Self-reported COVID-19 vaccination coverage in our study was high among HCWs working in close patient contact. We believe that prioritising HCWs for COVID-19 vaccinations and widespread vaccine availability, as well as generally low levels of vaccine hesitancy and typically high seasonal influenza vaccination coverage among our study population, were the main reasons for this result. Protection against COVID-19 provided by the vaccines was clearly the most important reason for having the COVID-19 vaccine; concerns about possible adverse reactions to the vaccine was the main reason for declining it. If other similar pandemics emerge in the future, ensuring the vaccines’ safety and efficacy will be essential in achieving high vaccination coverage among HCWs. Most of the HCWs agreed that all HCWs working in close patient contact should be vaccinated against COVID-19 – mandatory vaccination could increase vaccination coverage. However, our study indicated, that high vaccination coverage among HCWs can be achieved rapidly without the need for mandatory policies, at least during pandemics.

## Supplemental Material

sj-docx-1-sjp-10.1177_14034948231203779 – Supplemental material for COVID-19 vaccination among health care workers in Finland: coverage, perceptions and attitudes Supplemental material, sj-docx-1-sjp-10.1177_14034948231203779 for COVID-19 vaccination among health care workers in Finland: coverage, perceptions and attitudes by Aleksi Hämäläinen, Riitta-Liisa Patovirta, Sakari Vuorinen, Jaana Leppäaho-Lakka, Sanna Kilpinen, Jennifer Sieberns, Eija Ruotsalainen, Irma Koivula and Sari Hämäläinen in Scandinavian Journal of Public Health
