# Supplementary material for: COVID-19 vaccination among health care workers in Finland: coverage, perceptions and attitudes
Source: Scand J Public Health. 2024 Jan 3;52(3):309–15. doi: 10.1177/14034948231203779 (PMC11067404; doi:10.1177/14034948231203779)
Supplement: sj-docx-1-sjp-10.1177_14034948231203779 – Supplemental material for COVID-19 vaccination among health care workers in Finland: coverage, perceptions and attitudes [file sj-docx-1-sjp-10.1177_14034948231203779.docx]

**Supplementary table 1** COVID-19 vaccination status and opinions about COVID-19 by gender. In statistical analysis, males were compared to females and each age group was compared to other age groups combined. P-values were calculated with chi-square (χ2) test and Fisher’s exact test. * = calculated with Fischer’s Exact Test

|  | Males    n = 370  n (% in the category) | Females    n = 1467  n (% in the category) | P-value | <30 years old    n = 232  n (% in the category) | P-value | 30-50 years old    n = 968  n (% in the category) | P-value | >50 years old    n = 637  n (% in the category) | P-value |
| --- | --- | --- | --- | --- | --- | --- | --- | --- | --- |
| Have you taken at least one COVID-19 vaccine? |  |  |  |  |  |  |  |  |  |
| Yes | 361 (97.6) | 1423 (97.0) | 0.560 | 222 (95.7) | 0.165 | 931 (96.2) | 0.011 | 631 (99.1) | <0.001 |
| No | 9 (2.4) | 44 (3.0) |  | 10 (4.3) |  | 37 (3.8) |  | 6 (0.9) |  |
| Do you consider COVID-19 as a serious disease to yourself? |  |  |  |  |  |  |  |  |  |
| Yes | 194 (52.4) | 859 (58.6) | 0.033 | 90 (38.8) | <0.001 | 503 (52.0) | <0.001 | 460 (72.2) | <0.001 |
| No | 116 (31.4) | 324 (22.1) | <0.001 | 92 (39.7) | <0.001 | 264 (27.3) | <0.001 | 84 (13.2) | <0.001 |
| Uncertain | 60 (16.2) | 284 (19.4) | 0.094 | 50 (21.6) | 0.138 | 201 (20.8) | 0.018 | 93 (14.6) | <0.001 |
| Do you consider COVID-19 as a serious disease to persons belonging to risk groups? |  |  |  |  |  |  |  |  |  |
| Yes | 365 (98.6) | 1450 (98.8) | 0.761 | 230 (99.1) | 0.615* | 953 (98.5) | 0.143 | 632 (99.2) | 0.236 |
| No | 2 (0.5) | 7 (0.5) | 0.569* | 1 (0.4) | 0.683* | 7 (0.7) | 0.119* | 1 (0.2) | 0.125* |
| Uncertain | 3 (0.8) | 10 (0.7) | 0.504* | 1 (0.4) | 0.497* | 8 (0.8) | 0.522 | 4 (0.6) | 0.510* |
| Has the type of the vaccine influenced to your decision to take the vaccine? |  |  |  |  |  |  |  |  |  |
| Yes | 66 (17.8) | 363 (24.7) | 0.005 | 66 (28.4) | 0.050 | 213 (22.0) | 0.149 | 150 (23.5) | 0.886 |
| No | 288 (77.8) | 1007 (68.6) | <0.001 | 146 (62.9) | 0.007 | 692 (71.5) | 0.325 | 457 (71.7) | 0.393 |
| Uncertain | 16 (4.3) | 97 (6.6) | 0.102 | 20 (8.6) | 0.068 | 63 (6.5) | 0.502 | 30 (4.7) | 0.144 |
| Do you support that all HCWs working in patient contact should be vaccinated against COVID-19? |  |  |  |  |  |  |  |  |  |
| Yes | 265 (71.6) | 976 (66.5) | 0.062 | 122 (52.6) | <0.001 | 621 (64.2) | 0.001 | 498 (78.2) | <0.001 |
| No | 67 (18.1) | 303 (20.7) | 0.275 | 68 (29.3) | <0.001 | 230 (23.8) | <0.001 | 72 (11.3) | <0.001 |
| Uncertain | 38 (10.3) | 188 (12.8) | 0.105 | 42 (18.1) | 0.004 | 117 (12.1) | 0.766 | 67 (10.5) | 0.090 |
